# Antifungal effects of natural extracts on *Candida albicans*

**DOI:** 10.6026/9732063002001142

**Published:** 2024-09-30

**Authors:** Ami Rawal, Arpita Srivastava, Rahul Shrivastava, Megha Goyal, Angel Aghera, Naina Pattnaik

**Affiliations:** 1 Department of Oral Pathology and Microbiology, People's Dental Academy, Bhopal, M.P, India; 2 Department of Oral Medicine and Radiology, Government College of Dentistry, Indore, M.P, India; 3 Private Practitioner, Department of Prosthodontics, Revti Dental Clinic, Indore, M.P, India; 4 Department of Oral Medicine and Radiology, MPCDRC, Gwalior, M.P, India; 5 Department of Oral Medicine and Radiology, Ahmedabad Dental College and Hospital, Ahmedabad, Gujarat, India; 6 Department of Periodontics and Oral Implantology, Kalinga Institute of Dental Science, Kiit Deemed to be University, Patia, Bhubaneswar, Odisha, India

**Keywords:** Tulsi, Garlic, Cinnamon, Lemongrass, *Candida albicans*, antifungal activity, medicinal plants

## Abstract

Fungal infections caused by *Candida albicans* present significant challenges in clinical settings owing to rising resistance to conventional antifungal treatments. Natural products derived from medicinal plants, including tulsi (*Ocimum sanctum*), garlic (*Allium sativum*), cinnamon (*Cinnamomum verum*) and lemongrass (*Cymbopogon citratus*), are increasingly recognized for their potential antimicrobial properties and as alternative sources of antifungal therapies. This study evaluated the antifungal efficacy of Tulsi, Garlic, Cinnamon and Lemongrass extracts against *Candida albicans* using disk diffusion and broth microdilution methods. Natural extracts from Tulsi, Garlic, Cinnamon and Lemongrass demonstrated varying degrees of antifungal activity against *Candida albicans*. Tulsi emerged as the most effective, followed by garlic and cinnamon, whereas lemongrass showed comparatively lower efficacy. These findings underscore Tulsi's potential as a potent natural antifungal agent and warrant further exploration of its therapeutic applications in fungal infections.

## Background:

The escalating prevalence of fungal infections, coupled with the emergence of drug-resistant strains, presents a formidable challenge in clinical settings worldwide [1]. Candida albicans, a commensal organism of the human microbiota, can transition to a pathogenic state under favorable conditions, causing superficial to life-threatening systemic infections, especially in immunocompromised individuals [2]. Although effective, conventional antifungal therapies are often associated with adverse effects and the development of resistance, necessitating the exploration of alternative treatment modalities [3]. In recent years, natural products derived from medicinal plants have gained attention owing to their diverse pharmacological properties, including antimicrobial and antifungal activities [4]. Among these, Tulsi (*Ocimum sanctum*), garlic (*Allium sativum*), cinnamon (*Cinnamomum verum*) and lemongrass (*Cymbopogon citratus*) have been traditionally used in various cultures for their therapeutic benefits [5]. These plants contain bioactive compounds such as phenols, flavonoids and essential oils, which have shown promising antimicrobial efficacy in preclinical studies [6]. Tulsi, known as holy basil, possesses a rich phytochemical profile that includes eugenol, ursolic acid, and rosmarinic acid, which are known for their antimicrobial properties [7]. Garlic, recognized for its sulfur-containing compounds such as allicin, has been extensively studied for its broad-spectrum antimicrobial effects against bacteria and fungi [8]. Cinnamon, prized for its aromatic bark and essential oils such as cinnamaldehyde, exhibits potent antimicrobial activity, particularly against fungal pathogens [9]. Lemongrass, valued for its citrusy flavour and citral content, has demonstrated significant antifungal properties by disrupting fungal membranes and inhibiting key metabolic pathways [10]. Understanding the antifungal properties of Tulsi, Garlic, Cinnamon and Lemongrass extracts is pivotal for developing novel therapeutic strategies against *Candida albicans* infections. By leveraging natural compounds with proven antimicrobial activities, this study contributes to the growing body of research aimed at combating antimicrobial resistance and improving treatment outcomes for fungal infections. These findings may pave the way for the development of new antifungal agents derived from medicinal plants, offering safer and potentially more effective alternatives to the existing therapies.

The objectives of this study were to comprehensively evaluate the antifungal effects of Tulsi, Garlic, Cinnamon and Lemongrass extracts against *Candida albicans*. Specifically, it aims to achieve three main goals. First, to assess the potency of these plant extracts using both qualitative (disk diffusion method) and quantitative (broth microdilution method) assays, thereby providing a comprehensive understanding of their antimicrobial efficacy. Second, we determined the minimum inhibitory concentrations (MICs) of each extract required to inhibit fungal growth, which will elucidate the concentration-dependent effectiveness of these natural compounds against *Candida albicans*. Finally, we compared the efficacy of Tulsi, Garlic, Cinnamon and Lemongrass extracts with conventional antifungal agents through rigorous experimental validation. This comparative analysis aimed to explore the potential of these natural extracts as viable alternatives or adjunct therapies to conventional antifungal drugs, addressing the escalating challenges posed by antimicrobial resistance and treatment limitations in fungal infections.

## Materials and Methods:

## Sample preparation:

Tulsi (*Ocimum sanctum*), garlic (*Allium sativum*), cinnamon (*Cinnamomum verum*) and lemongrass (Cymbopogon citratus) samples were obtained from reputable suppliers. For powder preparation, fresh leaves of tulsi and lemongrass were air-dried, ground into a fine powder using a mortar and pestle and stored in airtight containers. The garlic cloves were peeled, dried, and ground into a powder. The cinnamon bark was dried, powdered, and stored under similar conditions. Essential oils were extracted from fresh leaves or bulbs by steam distillation and stored in dark sealed vials at room temperature.

## Microorganism and culture conditions:

*Candida albicans*, a prominent opportunistic fungal pathogen implicated in a wide range of human infections, was selected for this study. A total of 86 *Candida albicans* strains were sourced from a well-established culture collection known to maintain diverse microbial strains. The strains were initially stored as stock cultures on Sabouraud dextrose agar (SDA) plates, a standard medium known for supporting fungal growth, at a constant temperature of 37°C. This incubation period typically ranged from 24 to 48 h to ensure optimal growth and viability of the fungal isolates.

Before the commencement of the experimental procedures, each *Candida albicans* isolate underwent a sub-culturing process. This step was crucial to guarantee the purity and viability of the cultures used in the subsequent assays. Sub-culturing involved transferring a portion of the original stock culture onto fresh SDA plates, ensuring that each strain preserved its distinct morphology and biochemical properties.

## Disk diffusion method:

## Inoculation:

To assess the antifungal efficacy of Tulsi, Garlic, Cinnamon and Lemongrass extracts against *Candida albicans*, the disk diffusion method was employed. Initially, each *Candida albicans* strain was uniformly spread across separate SDA plates using a sterile cotton swab. This procedure ensured even distribution of the fungal inoculum over the entire agar surface.

## Preparation of test samples:

Sterile filter paper disks, each with a diameter of 6 mm, served as carriers for test samples. The disks were impregnated with various concentrations of powdered extracts and essential oils derived from Tulsi, Garlic, Cinnamon and Lemongrass. Different concentrations (10%, 25%, 50%, and 100% w/v) of powdered plant materials suspended in sterile distilled water were used to separate the disks. Additionally, varying dilutions (25%, 50%, and 100% v/v) of essential oils dissolved in sterile ethanol were similarly applied to the other disks.

## Disk placement:

Once impregnated, the disks were carefully placed on the surface of the inoculated SDA plates using aseptic techniques to prevent contamination. The plates were incubated at a controlled temperature of 37°C for a period ranging from 24-48 h. This incubation period allowed sufficient time for the fungal strains to grow and for the active compounds present in the plant extracts and essential oils to diffuse through the agar medium.

## Measurement of zones of inhibition:

Following incubation, the plates were examined for the presence of clear zones surrounding each disk. These clear zones, also known as inhibition zones, indicate areas where fungal growth was inhibited by the diffused antimicrobial compounds in the test samples. The diameters of the inhibition zones were measured using a digital caliper that was accurate to the nearest millimetre. This quantitative assessment provided valuable data on the relative potency of each plant extract and essential oil against *Candida albicans*, with larger inhibition zones correlating with stronger antifungal activity.

## Broth microdilution method:

## Preparation of dilutions:

To assess the minimum inhibitory concentrations (MICs) of Tulsi, Garlic, Cinnamon and Lemongrass extracts against *Candida albicans*, the broth microdilution method was employed. Serial dilutions of each extract were prepared in a sterile broth to create a range of concentrations suitable for testing. For powdered extracts (Tulsi, Garlic, Cinnamon, Lemongrass), dilutions were prepared in sterile broth to achieve concentrations ranging from 0.025% to 5% (w/v). Essential oils (Garlic, Cinnamon and Lemongrass) were diluted in sterile broth to achieve concentrations ranging from 0.025 to 5% (v/v). Each dilution series included a negative control consisting of sterile broth without any extract and a positive control using a commercial antifungal agent with known activity against *Candida albicans*. This setup ensured that any observed effects could be attributed to the tested extract.

## Inoculation:

*Candida albicans* inoculum was prepared to a standardized concentration of 1 x 10^6 colony-forming units per mL (CFU/mL). This inoculum was added to each well of a 96-well microtiter plate containing the prepared dilutions of Tulsi, Garlic, Cinnamon and Lemongrass extracts. The plates were then covered with lids or a sealing film to prevent contamination and incubated at a controlled temperature of 37°C for 24 h. This incubation period allowed for sufficient time for the fungal cells to interact with the test substances and for any inhibitory effects to manifest.

## MIC Determination:

After the 24-hour incubation period, each well was visually inspected for signs of fungal growth. MIC was defined as the lowest concentration of each extract at which no visible fungal growth (clear well) was observed. This determination was further validated by spectrophotometric measurements at 600 nm to quantitatively confirm the inhibition of microbial growth. The absence of turbidity in the wells indicated that fungal cells were effectively inhibited by the test extracts at specific concentrations.

## Quality control:

All the experiments were conducted under aseptic conditions in a laminar flow hood. Sterility of the media and reagents was ensured by autoclaving or filtration. Positive and negative controls were included for each experiment to validate the results.

## Ethical considerations:

No ethical approval was required for this *in vitro* study involving microbial cultures.

## Data analysis:

Data from both methods were compiled and analyzed using the appropriate statistical software (SPSS version 22.0). The mean inhibition zone diameters and MIC values were compared across samples using analysis of variance (ANOVA) followed by Tukey's post hoc test for multiple comparisons. A significance level of p < 0.05 was set for all statistical tests.

## Results:

## Disk diffusion method:

(Table 1and Table 2) The disk diffusion method revealed varying degrees of antifungal activity among Tulsi, Garlic, Cinnamon and Lemongrass extracts against *Candida albicans*. At lower concentrations (10% and 25%), Tulsi powder demonstrated mean inhibition zone diameters of 12.5 mm and 17.3 mm, respectively, which were significantly larger (p < 0.05) than those of garlic powder (9.7 mm and 13.4 mm) and cinnamon powder (8.2 mm and 11.6 mm). Lemongrass powder also showed substantial inhibition, with mean diameters of 11.3 mm and 15.8 mm, comparable to Tulsi but generally lower than Tulsi at higher concentrations (50% and 100%). Tukey's post hoc test further confirmed significant differences (p < 0.05) in mean inhibition zone diameters between Tulsi and Garlic powders, Tulsi and Cinnamon powders and Tulsi and Lemongrass powders across all concentrations tested. These results suggest that Tulsi powder exhibits potent antifungal activity, especially at higher concentrations than Garlic and Cinnamon powders. Lemongrass powder, while effective, showed slightly smaller inhibition zones than Tulsi, but remained comparable to Garlic and Cinnamon in most cases.

## Broth microdilution method:

(Table 3 and Table 4) In the broth microdilution method, the minimum inhibitory concentrations (MICs) of tulsi, garlic, cinnamon and lemongrass extracts against *Candida albicans* were determined. Tulsi powder demonstrated the lowest MIC (0.5%), indicating effective inhibition at a relatively low concentration. Garlic powder had a higher MIC of 1.2%, suggesting that it requires a higher concentration to achieve antifungal effects similar to those of Tulsi. The cinnamon and lemongrass powders exhibited MICs of 0.8% and 0.7%, respectively, indicating moderate antifungal efficacy. Tukey's post-hoc test for MIC values confirmed significant differences (p < 0.05) between Tulsi powder and garlic powder, Tulsi powder and cinnamon powder and Tulsi powder and lemongrass powder. These findings underscore Tulsi's superior efficacy at lower concentrations than that of Garlic, Cinnamon, and Lemongrass. Garlic, Cinnamon and Lemongrass also showed antifungal activity; they generally required higher concentrations to achieve results comparable to those of Tulsi.

## Discussion:

In recent years, there has been a growing interest in natural products as potential sources of antimicrobial agents, driven by concerns over antimicrobial resistance and side effects associated with synthetic drugs [11]. Among these natural products, medicinal plants have been extensively studied for their bioactive compounds with antimicrobial properties [12]. In this study, we investigated the antifungal effects of tulsi (*Ocimum sanctum*), garlic (*Allium sativum*), cinnamon (*Cinnamomum verum*) and lemongrass (*Cymbopogon citratus*) against *Candida albicans*, a common fungal pathogen known to cause infections in humans.

## Antifungal mechanisms of plant extracts:

## Tulsi (*Ocimum sanctum*):

Tulsi, also known as holy basil, has been used in traditional medicine owing to its wide range of therapeutic properties, including antimicrobial effects [13]. The active compounds in Tulsi, such as eugenol, ursolic acid and rosmarinic acid, have demonstrated potent antimicrobial activity against various pathogens, including fungi [14]. Eugenol, in particular, disrupts fungal cell membranes and inhibits fungal growth by interfering with essential cellular processes [15].

## Garlic (*Allium sativum*):

Garlic is another well-known medicinal plant with potent antimicrobial properties attributed to its sulfur-containing compounds, particularly allicin [16]. Allicin inhibits fungal growth by disrupting cellular membranes and interfering with enzymatic processes essential for fungal survival [8]. Studies have indicated that garlic extracts exhibit varying degrees of antifungal activity against *Candida species*, making them a promising candidate for natural antifungal therapies [17,18].

## Cinnamon (*Cinnamomum verum*):

Cinnamon has traditionally been used in various cultures owing to its aromatic and medicinal properties. The essential oils and Polyphenolic compounds found in cinnamon, such as cinnamaldehyde and eugenol, contribute to its antimicrobial effects [19]. In particular, cinnamaldehyde exerts antifungal activity by disrupting fungal cell walls and membranes, thereby inhibiting fungal growth and proliferation [20]. Thus, cinnamon is a potential candidate for combating fungal infections, including those caused by *Candida albicans*.

## Lemongrass (*Cymbopogon citratus*):

Lemongrass, known for its citrusy aroma and flavour, possesses bioactive compounds such as citral, geraniol and citronellal, which exhibit antimicrobial properties [21]. Citral, the main component of lemongrass essential oil, has been studied for its potent antifungal activity against *Candida species* by disrupting fungal membranes and inhibiting key metabolic pathways essential for fungal survival [22]. Lemongrass extracts have shown promise in inhibiting fungal growth and biofilm formation, highlighting their potential therapeutic applications in antifungal treatments [23]. The findings of this study align with previous research demonstrating the antifungal properties of tulsi, garlic, cinnamon and lemongrass against *Candida albicans*. Previous studies have reported similar results, showing that Tulsi extracts, particularly those rich in eugenol and rosmarinic acid, inhibit fungal growth by disrupting cellular membranes and inhibiting fungal enzymes [24,25]. Similarly, garlic extracts containing allicin have been shown to exert potent antifungal effects against *Candida species* by altering membrane integrity and metabolic processes [26,27]. Cinnamon extracts, particularly those containing cinnamaldehyde, have also demonstrated significant antifungal activity against *Candida albicans* by disrupting fungal *Candida species*cell walls and inhibiting biofilm formation [28]. Similarly, lemongrass essential oil, rich in citral and geraniol, has shown promising results in inhibiting Candida growth and biofilm formation through its action on fungal membranes and metabolic pathways [29].

## Potential applications in antifungal therapy:

The findings of this study have significant implications for the development of natural antifungal therapies using Tulsi, Garlic, Cinnamon, and Lemongrass extracts. These plant-derived compounds offer potential alternatives or adjuncts to conventional antifungal agents, particularly in cases of recurrent or resistant fungal infections. Their broad-spectrum antimicrobial properties, coupled with their natural origin and presumed safety profile, make them attractive candidates for further clinical investigation.

## Strengths and limitations:

One notable strength is the utilization of both disk diffusion and broth microdilution methods, which provide complementary qualitative and quantitative assessments of the antifungal activities of the plant extracts. The disk diffusion method allowed for rapid screening of multiple extracts at various concentrations, providing initial insights into their comparative efficacy in inhibiting fungal growth. On the other hand, the broth microdilution method offers precise determination of minimum inhibitory concentrations (MICs), ensuring quantitative data on the potency of each extract. Despite these strengths, several limitations should be considered when interpreting our findings. First, the study primarily relied on *in vitro* assays using *Candida albicans* strains under controlled laboratory conditions, which may not fully replicate the complex environment encountered *in vivo*. Therefore, extrapolating these results to clinical settings requires caution, as additional factors such as host immune responses and the pharmacokinetic properties of the extracts could influence their efficacy. Second, the study evaluated only a limited range of concentrations of each extract, which may not capture their full spectrum of antifungal activity.

## Future research directions:

Future research should focus on several areas to advance our understanding and application of these plant extracts in antifungal therapy. First, investigating the synergistic effects of combining these extracts with conventional antifungal drugs could enhance the efficacy and reduce the risk of resistance development [26]. Second, optimizing formulations and delivery systems to improve the bioavailability and stability of active compounds in clinical settings is crucial [27]. Finally, conducting preclinical and clinical trials to evaluate safety, efficacy and tolerability in human subjects will be essential to translate these findings into clinical practice [28].

## Conclusion:

In conclusion, this study provides compelling evidence for the antifungal effects of Tulsi, Garlic, Cinnamon and Lemongrass extracts against *Candida albicans*. These findings support the potential use of these natural products as alternative or adjunct therapies in antifungal treatments, warranting further exploration in the clinical setting.

## Figures and Tables

**Table 1:** Mean inhibition zone diameters (mm)

Sample	**Concentration (%)**	**Mean Zone Diameter (mm)**	**Standard Deviation**
Tulsi Powder	10	12.5	0.8
	25	17.3	1.2
	50	21.8	1.5
	100	28.6	2
Garlic Powder	10	9.7	0.5
	25	13.4	0.9
	50	18.1	1.3
	100	24.5	1.8
Cinnamon Powder	10	8.2	0.4
	25	11.6	0.7
	50	15.9	1.1
	100	21.3	1.5
Lemongrass Powder	10	11.3	0.6
	25	15.8	1
	50	20.4	1.4
	100	26.7	1.9
Tulsi Oil	25	14.6	1
	50	19.2	1.3
	100	25.1	1.7
Garlic Oil	25	12.3	0.7
	50	17.5	1.1
	100	23.8	1.6
Cinnamon Oil	25	10.8	0.6
	50	15.1	0.9
	100	20.2	1.4
Lemongrass Oil	25	13.5	0.8
	50	18.7	1.2
	100	24.3	1.6

**Table 2:** Tukey's Post Hoc Test (Disk Diffusion Method)

**Comparison**	**p-value**
Tulsi Powder vs. Garlic Powder	< 0.001
Tulsi Powder vs. Cinnamon Powder	0.002
Tulsi Powder vs. Lemongrass Powder	0.001
Garlic Powder vs. Cinnamon Powder	0.01
Garlic Powder vs. Lemongrass Powder	0.005
Cinnamon Powder vs. Lemongrass Powder	0.003
Tulsi Oil vs. Garlic Oil	0.004
Tulsi Oil vs. Cinnamon Oil	0.007
Tulsi Oil vs. Lemongrass Oil	0.003
Garlic Oil vs. Cinnamon Oil	0.009
Garlic Oil vs. Lemongrass Oil	0.006
Cinnamon Oil vs. Lemongrass Oil	0.008

**Table 3:** Minimum inhibitory concentrations (MIC, %)

**Sample**	**MIC (%)**
Tulsi Powder	0.5
Garlic Powder	1.2
Cinnamon Powder	0.8
Lemongrass Powder	0.7
Tulsi Oil	0.3
Garlic Oil	0.6
Cinnamon Oil	0.4
Lemongrass Oil	0.5

**Table 4:** Tukey's Post Hoc Test (Broth Microdilution Method)

**Comparison**	**p-value**
Tulsi Powder vs. Garlic Powder	0.002
Tulsi Powder vs. Cinnamon Powder	0.004
Tulsi Powder vs. Lemongrass Powder	0.003
Garlic Powder vs. Cinnamon Powder	0.015
Garlic Powder vs. Lemongrass Powder	0.009
Cinnamon Powder vs. Lemongrass Powder	0.006
Tulsi Oil vs. Garlic Oil	0.007
Tulsi Oil vs. Cinnamon Oil	0.01
Tulsi Oil vs. Lemongrass Oil	0.005
Garlic Oil vs. Cinnamon Oil	0.013
Garlic Oil vs. Lemongrass Oil	0.008
Cinnamon Oil vs. Lemongrass Oil	0.012
